# Impact of temperatures on malaria incidence in vulnerable regions of Pakistan: empirical evidence and future projections

**DOI:** 10.1017/S0950268825000111

**Published:** 2025-01-31

**Authors:** Syeda Hira Fatima, Farrah Zaidi, Javeria Rafiq, Dinesh Bhandari, Asad Ali, Peng Bi

**Affiliations:** 1 Global Ecology, Partuyarta Ngadluku Wardli Kuu, College of Science and Engineering, Flinders University, Adelaide, South Australia, Australia; 2 College of Medicine and Public Health, Flinders University, Adelaide, South Australia, Australia; 3 School of Public Health, The University of Adelaide, Adelaide, South Australia, Australia; 4 Zoology Department, Peshawar University, Peshawar, Khyber Pakhtunkhwa, Pakistan; 5 School of Nursing and Midwifery, Monash University, Monash, Victoria, Australia; 6 Monash Health and Climate Initiative, Monash University, Monash, Victoria, Australia; 7 Statistics Department, Institute of Space Technology, Islamabad, Pakistan

**Keywords:** climate, epidemiology, heat, low and middle-income countries, malaria

## Abstract

Malaria remains a major health challenge in developing countries, with climate change intensifying its impact. Pakistan is among the most vulnerable nations. This study examines the relationship between temperature and malaria cases in two highly affected districts, Bannu and Lakki Marwat, to inform climate-adaptive interventions.

We analyzed monthly malaria cases (2014–2022) from the Integrated Vector Control/Malaria Control Program in Khyber Pakhtunkhwa, combined with gridded meteorological data from Copernicus ERA5-Land. Time-series analysis using distributed lag nonlinear models and quasi-Poisson regression was applied to assess the associations.

The findings suggest that as temperatures exceed 22.4°C, malaria transmission increases by 9 to 10% for every 1°C rise in both districts. In Bannu, up to 39.8% of reported malaria cases could be attributed to heat, while in Lakki Marwat, 54.1% of cases were attributable to heat. Under high emission scenarios, heat-related malaria cases could increase by 0.8 to 3.5% by the 2060s. Relationship between temperature and malaria transmission is complex and is influenced by environmental factors such as precipitation and humidity.

Given Pakistan’s limited healthcare infrastructure, addressing climate-driven malaria risks is urgent. Recent severe floods and malaria surges highlight the need for climate adaptation measures and strengthened healthcare systems to enhance community resilience.

## Key findings

- For every 1 °C rise above optimal temperature, malaria risk increases by 9–10%.
- About 39.8–54.1% of malaria cases are attributable to heat.
- Heat-related cases of malaria are projected to increase by 3.5% by 2060s in remote regions of Pakistan.

## Introduction

Malaria stands as a prominent cause of morbidity and mortality in many developing nations. As of 2015, which served as the baseline year for the Global Technical Strategy for Malaria 2016–2030 (GTS), an estimated 231 million cases of malaria were reported [1]. By 2022, there was a notable increase with an estimated 249 million cases documented in 85 malaria-endemic countries and regions (including French Guiana), marking an increase of five million cases from 2021. Key contributors to this surge included Pakistan (+2.1 million), Ethiopia (+1.3 million), Nigeria (+1.3 million), Uganda (+597000), and Papua New Guinea (+423000) [1]. The ongoing threat of malaria affects over two billion individuals, including both travellers and residents in endemic areas, resulting in an annual toll of about 608000 deaths as of 2022 [1, 2].

In recent years, the profound impact of climate change on malaria has attracted significant attention due to its potential to exacerbate the disease burden and alter transmission dynamics [3]. While climate change affects various aspects of malaria transmission, including vector abundance and pathogen development, rising temperatures are of paramount concern. Rising temperatures can expand the geographic range and local abundance of malaria vectors, *Anopheles* mosquitoes, which thrive in warm climates [4]. Furthermore, warmer temperatures accelerate the development of malaria parasites within mosquitoes and shorten the incubation period of the disease in humans, leading to an increased risk of transmission [5].

Many studies have incorporated climate change scenarios and geostatistical models to explain and project future malaria incidence locally and globally. Various process-based or mechanistic models have been proposed for the intricate and non-linear weather-driven *Anopheles* lifecycle and malaria transmission dynamics. However, these models have yielded somewhat divergent findings regarding the optimal temperatures for transmission and the potential impact of rising temperatures and other extreme weather events on the distribution of malaria. The projections differ: some suggest a significant increase in malaria-susceptible areas, while others predict a shift in the geographical range of the disease [4].

Pakistan remains the most susceptible country to the repercussions of malaria, with the disease ranking as the fourth-largest cause of death among infectious diseases. Pakistan and other countries in the WHO Eastern Mediterranean Region, such as Afghanistan, Somalia, Sudan, and Yemen, jointly account for 95% of all malaria cases reported in the region [6]. Pakistan is considered to be hyperendemic for malaria, and the pooled malaria prevalence is estimated at 23.3% [7]. Malaria is most prevalent in Khyber Pakhtunkhwa (KPK) and Balochistan provinces [7]. *An. culicifacies, An. stephensi*, *An. subpictus, and An. superpictus* are the primary vectors for malaria and have been reported to be endemic in the region since the early 1900s [8–9]. *Plasmodium vivax* (prevalence rate: 79.13%) and *P. falciparum* (prevalence rate: 16.29%) are the predominant malaria parasite species [6–7]. Pakistan’s ecological conditions, characterized by a monsoon-fed agricultural landscape with flat terrain towards the south, provide ideal settings for malaria transmission. Temperatures from September to December and April to May frequently range between 20 °C and 30 °C, facilitating mosquito breeding, while rainfall creates stagnant water pools essential for larvae development. Higher humidity levels further enhance malaria transmission.

Over the years, entomological studies in Pakistan have elucidated the vector ecology of *Anopheles* mosquitoes, and the prevalence and epidemiology of malaria [8–12]. The government has launched malaria control efforts to curb the disease; however, Pakistan faces challenges in reducing malaria incidence. These challenges could be partly attributed to climate change, particularly the irregular temperature and rainfall patterns in recent years [7]. Recurrent massive flooding events have exacerbated these challenges by creating additional breeding habitats for mosquitoes, thereby facilitating the spread of mosquito-borne diseases such as malaria and dengue [12].

Despite the pressing nature of this issue, there is a notable scarcity of empirical evidence on how climate change specifically affects malaria in low and middle-income countries like Pakistan. This scarcity creates a significant obstacle to fully understanding the extent of the problem and implementing effective mitigation and adaptation strategies.

Recently, after catastrophic flooding in Pakistan in 2022 and the subsequent increase in malaria incidence, it has become imperative to estimate the climate change-attributed increase in the risk of malaria. Understanding the role of climate change in the resurgence of malaria will guide policy decisions aimed at achieving the ambitious goal of a ‘Malaria-Free Pakistan by 2035’ [13].

This study aims to provide empirical evidence of the influence of climate change, using high temperature as an indicator, on malaria incidence in two highly vulnerable districts of Pakistan and project future risk estimates. This will lay a solid foundation for informing response strategies and addressing the uneven implementation of malaria interventions.

## Methods

### Study settings

**Figure 1.**
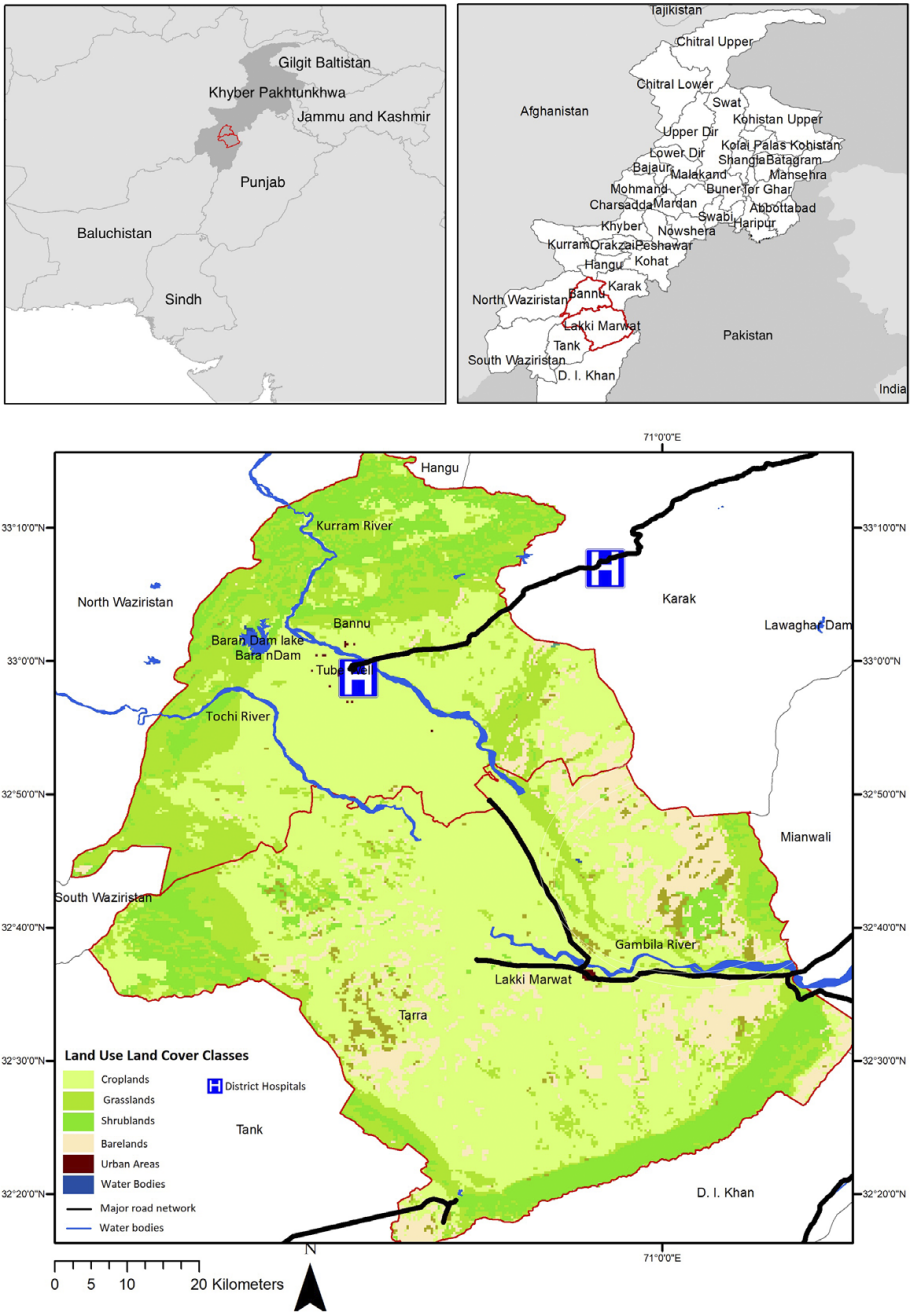
Study area map, depicting the predominant land cover characteristics of the region.

Bannu and Lakki Marwat districts are situated in the southern region of the KPK province in Pakistan (Figure 1). Bannu covers an area of 1227 sq. km with a population of 1167892, while Lakki Marwat spans 3296 sq. km and has a population of 876182 (Pakistan Bureau of Statistics, 2017). These districts are recognized as hyperendemic regions for malaria incidence in KPK, Pakistan [6]. Bannu district has been known for its high malaria prevalence since the British Raj [10]. In Bannu, *P. vivax* has a prevalence of 16.9% while *P. falciparum* has a prevalence of 2.3%. In Lakki Marwat, one study estimated the prevalence of *P. vivax* at 20.2% and *P. falciparum* at 0.2% [10]. Studies conducted in Pakistan provide evidence that malaria is predominantly a disease in rural areas (prevalence: 38.65%) where people live below the poverty line, as compared to urban areas (22.39%) [14]. Integrated Vector Control/Malaria Control Program KPK (IVC/MCP-KP) and Frontier Primary Health Care (FPHC) manage control interventions such as Indoor Residual Spraying (IRS), widespread bed net distribution, provision of bed nets to pregnant women during antenatal care, and community education campaigns. Antimalaria measures have been in practice since the colonial era, including fumigating and spraying against adult mosquitos resting in buildings, filling and draining breeding sites, treating them with oil or Paris Green, and, in the case of the smaller irrigation channels, completely drying them out once a week [10]. In terms of healthcare, in Pakistan, Chloroquine serves as the primary treatment for unconfirmed malaria, Chloroquine-Primaquine is recommended for *P. vivax*, and Artesunate/Sulfadoxine-Pyrimethamine (AS+SP) is employed for uncomplicated *P. falciparum* malaria, with severe cases or treatment failures addressed using Artesunate, Artemether, or Quinine [6].

The districts experience a semi-arid climate with hot and dry summers lasting seven months from April to October. June experiences maximum temperatures ranging from 42 °C to 45 °C, and the average annual temperature is 24.2 °C. The annual rainfall in Bannu and Lakki Marwat ranges from 120.0 to 270.0 mm, and rainfall is sporadic, occurring mainly during the monsoon season. Both districts are predominantly rural, characterized by cropland and shrubland cover. Sandstorms are periodic occurrences, particularly during May and June, affecting the entire area.

### Environmental data

Monthly gridded meteorological datasets were acquired for the period of 2014–2022 from Copernicus ERA5-Land, featuring variables such as mean temperature (°C), precipitation (mm), and dew point (°C), all at a resolution of 9x9 kilometres [15]. Relative humidity (%) was estimated using dew point and mean temperature [16]. These datasets were extracted for the two districts using centroid points.

### Malaria data

In KPK, Pakistan, lab-confirmed cases of malaria are administered by IVC/MCP-KP and FPHC. Malaria diagnosis is conducted using microscopy and Rapid Diagnostic Tests (RDTs), with microscopy being the gold standard. In areas where electricity and trained microscopists are limited, RDTs are primarily used for diagnosis. The RDTs are procured through international procurement from the World Health Organization (WHO) approved list of RDTs. The data at the health facility level is checked and verified by the district malaria team and further cross-checked at the provincial level to ensure credibility and reliability. One Quality Assurance Officer and a microscopist at the provincial level audit the health facilities to ensure accurate diagnosis and provide on-the-job training to malaria supervisors. Monthly aggregated records of malaria cases from 2014 to 2022 were obtained from IVC/MCP-KP, Directorate of General Health Services KPK. The data were already fully de-identified at the source, provided in aggregated form, and contained no individual-level patient information, ensuring there was no need for further anonymization. Specifically, the dataset included only the total number of positive malaria cases per month, disaggregated into *P. vivax* (PV) and *P. falciparum* (PF) cases, as well as the number of RDTs conducted. The use and publication of this aggregated, de-identified data were authorized through the issuance of a No Objection Certificate from the IVC/MCP-KP, Directorate of General Health Services KPK (NOC provided in Supplementary File).

### Statistical analysis

A time-series study design coupled with distributed lag non-linear models was utilized to assess the effects of ambient temperature on the risk of malaria transmission between 2014 and 2022 in the monsoon-fed semi-arid districts of Pakistan [17]. Monthly malaria datasets were integrated with hydrometeorological variables to explore the association between mean temperature and malaria. To model the effect of mean temperature on malaria cases, generalized linear models fitted with distributed lags were applied, utilizing a quasi-Poisson distribution, for each district. A linear threshold function was defined for the mean temperature at 22.4 °C based on evidence suggesting that the biting activity of malaria-transmitting mosquitoes is higher above 22.4 °C [18].

To capture the optimal conditions for malaria transmission, predictions and attributable fractions were estimated within the temperature range of 22.4–35.3°C. This range was selected to reflect the temperatures most conducive to mosquito activity and *Plasmodium* parasite development [3, 18]. The lag dimension was modelled using an unconstrained distributed lag function for up to a period of three months. Natural cubic splines with two degrees of freedom per year were used to adjust for confounders that change slowly over time. The logarithm of the population was used as an offset variable to account for differences in population sizes among observations. Relative humidity and precipitation were included as covariates, as these are important factors regulating the growth and incubation period of malaria vectors.

The final model was structured as follows:

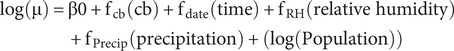

Where log(μ) is the expected monthly count of malaria cases, modelled as a linear combination of predictor variables, β0 is the intercept, and *f_cb_*(cb) represents the smooth function for the bi-dimensional relationship between temperature at each lag and across different lags. *f*_RH_ and *f*_Precip_ represent the smoothing functions for relative humidity and precipitation. Log (Population) is the offset term.

The estimate for the overall effect of mean temperature, *β^, was subsequently computed by summing all the contributions at different lags from the coefficients of the cross-basis.* This model assumed a log-linear relationship between the expected number of malaria cases and the mean temperature. The results were expressed as Relative Risk (RR) estimates, obtained by exponentiating the estimated β^, representing the percent change in the risk of malaria per degree increase in temperature.

### Estimation of attributable number (AN) and attributable fraction (AF)

Heat-related attributable numbers (AN) and attributable fractions (AF) of malaria cases were calculated following the method outlined by Gasparrini and Leone [19]. The AN quantifies the excess number of malaria cases attributable to heat exposure within the defined temperature range (22.4–35.3 °C). The AN was computed using the formula:

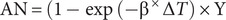

Where β*^* represents the coefficients derived from the DLNM, *ΔT* represents temperature levels during the observed or projected period, and Y represents the observed counts of malaria cases.

The AF was then calculated as the ratio of the AN to the total number of observed cases, expressed as a percentage:

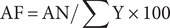

The AF represents the proportion of malaria cases attributable to heat exposure within the defined temperature range. To account for uncertainty in the coefficient estimates, 1000 iterations of Monte Carlo simulations were performed, generating confidence intervals for the AF estimates.

### Projections of future malaria burden

Projected future increases in mean temperature were obtained for two-time slices (2044–2052 and 2064–2072) under different climate change scenarios. The data were sourced from the World Bank’s Climate Change Knowledge Portal (CCKP) (Table 1) [20]. The heat-related AF was calculated under two Shared Socio-Economic Pathways (SSP2–4.5 and SSP2–8.5), using baseline data from 2014 to 2022. The derived temperature effect estimate (β^) was used to compute the RR and projected AN for future periods using the formula:

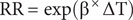

Where ΔT is substituted with the projected mean annual increase in temperature for each climate scenario. The projected AN and AF were calculated using the same approach as for the baseline period. In the absence of projected population data, we assumed no significant changes in the population when projecting the future burden of malaria.

**Table 1.** Characteristics of projected data obtained from World Bank’s CCKP

Projection coverage	Climate model used	Scenarios	Projection years	Projected increase in temperature (ΔT in °C)	Remarks	Data Source
Pakistan (KPK) 25X25 Km	Multi-Model Ensemble CMIP 6	SSP2–4.5	2040s	1.71	Average mean surface air temperature	https://climateknowledgeportal.worldbank.org/country/pakistan/climate-data-projections

All data preprocessing and analyses were conducted using R software (R 4.1.0), with the dlnm and mvmeta packages for model fitting [21]. Relevant R code used for the analyses and results is publicly available at https://doi.org/10.5281/zenodo.14751632.

## Results

### The relationship between environmental variables and malaria incidence

Descriptive statistics reveal that both Bannu and Lakki Marwat share similar climatic conditions. Bannu experiences average temperatures ranging from 9.45 °C to 35.05 °C, while Lakki Marwat’s average temperatures span from 10.79 °C to 35.35 °C. Humidity levels fluctuate between 19% and 74% in Bannu and 21% and 79% in Lakki Marwat (Table 2).

**Table 2.** Descriptive statistics on environmental variables and malarial incidence per 1000 RDTs in Bannu and Lakki Marwat

	Monthly Median (range)				
Districts	Mean Temperature (°C)	Humidity (%)	PV Prevalence Rates per 1000 RDT cases	PF Prevalence rate per 1000 RDT cases	Total Cases Prevalence rate per 1000 RDT cases
Bannu	24.60 (9.45, 35.05)	51.27 (19.57, 74.30)	144.93 (15.66, 1998.52)	2.87 (0.00, 47.30)	150.19 (16.65, 2008.89)
Lakki Marwat	25.70 (10.79, 35.35)	53.38 (21.58, 76.80)	92.81 (19.04, 229.30)	2.08 (0.26, 12.40)	95.36 (19.79, 232.35)

The calculated prevalence rates signify the occurrences of PV and PF per 1000 RDTs conducted within the studied population or timeframe. Predominantly, PV constitutes the majority of cases in both districts, with rates of 144.93 and 92.81 cases per 1000 RDTs in Bannu and Lakki Marwat, respectively. PF cases show prevalence rates of 2.87 and 2.08 per 1000 RDTs conducted in these respective regions (Table 2).

**Figure 2.**
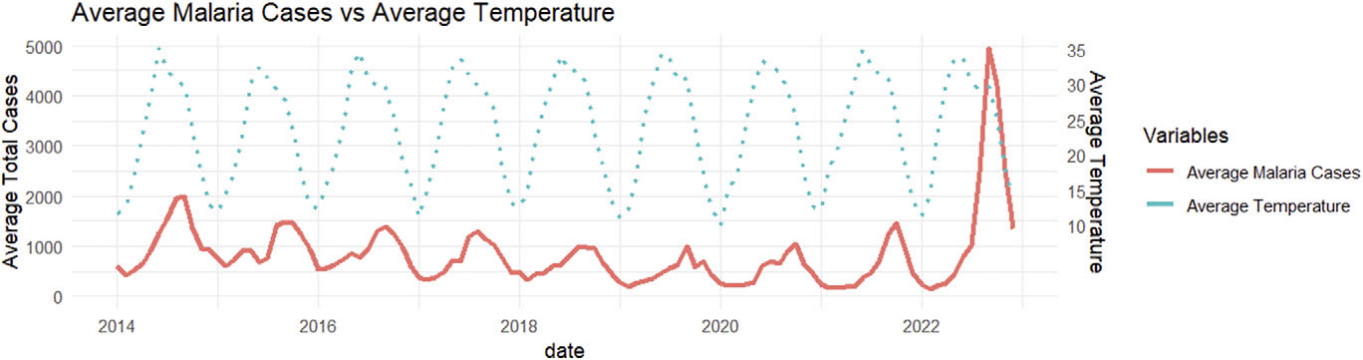
Average monthly malaria cases and temperature (οC).

**Figure 3.**
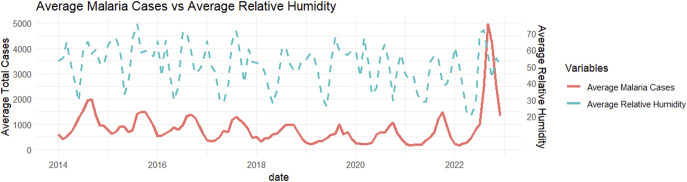
Average monthly malaria cases and humidity (%).

The monthly time-series distribution depicted in Figures 2 and 3 unveils the intricate relationship between weather patterns and malaria cases in Bannu and Lakki Marwat. Figures 2 and 3 indicate the presence of distinct seasonal patterns, characterized by alternating highs and lows in malaria cases. Notably, these fluctuations show a positive association between monthly variations in temperature (Spearman’s ρ = 0.34) and relative humidity (Spearman’s ρ = 0.28) with malaria cases throughout the region.

Average monthly trends of malaria, corresponding to variations in temperature and humidity (Figures 2 and 3), underscore a noteworthy trend observed from 2014 onwards – an evident decline in reported malaria cases. However, a significant surge becomes apparent in the year 2022.

**Figure 4.**
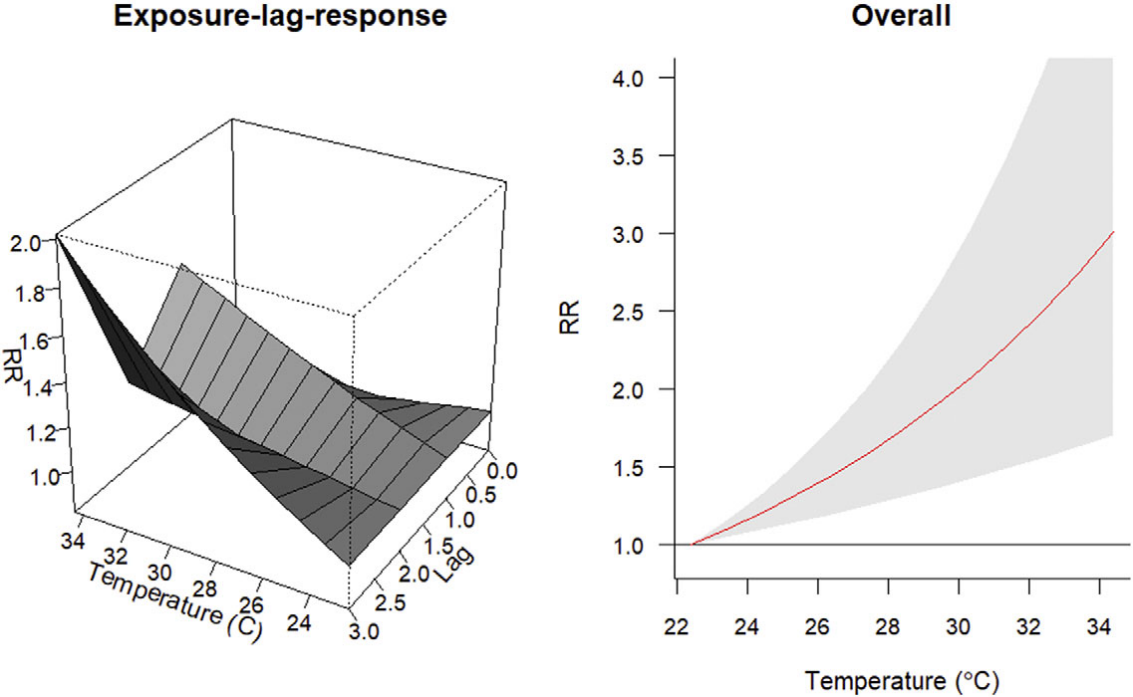
The exposure-response association between monthly malarial cases and mean temperature in Bannu. A. Exposure-lag response. B. Overall cumulative relative risk (RR).

**Figure 5.**
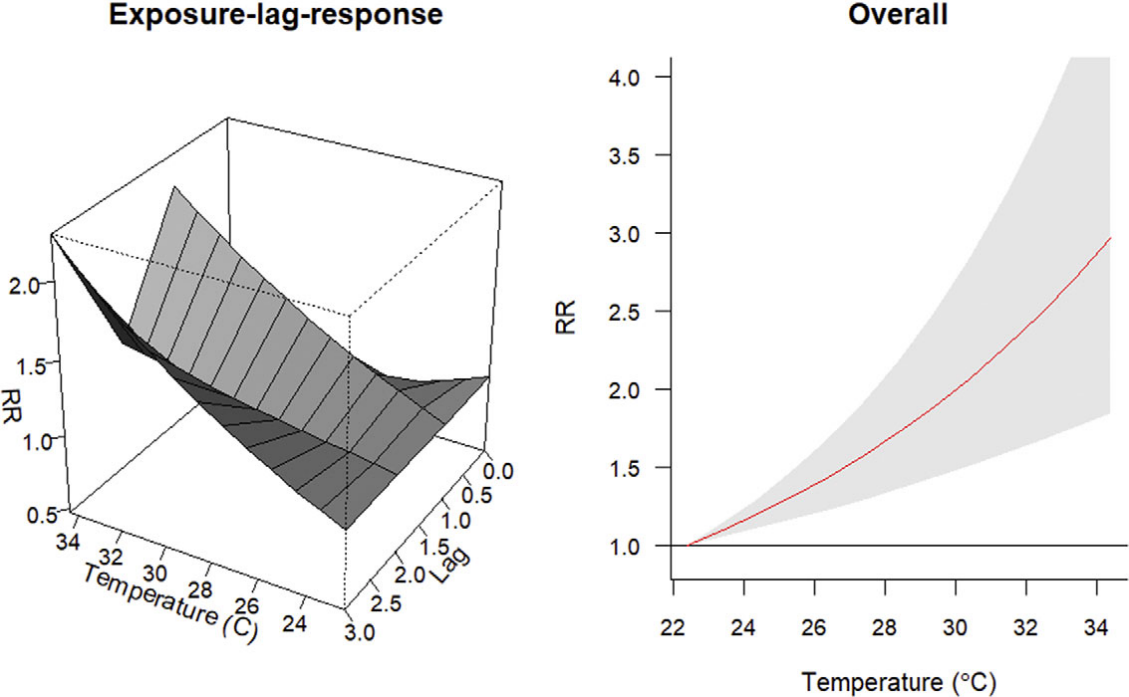
The exposure-response association between monthly malarial cases and mean temperature in Lakki Marwat. A. Exposure-lag response. B. Overall cumulative relative risk (RR).

In both Bannu and Lakki Marwat, the risk of malaria cases increased with rising temperatures above 22.4 °C (Figures 4 and 5). In Bannu, for every 1 °C increase above this threshold, there was a 9.87% (95%CI: 4.86, 15.12) rise in the risk of malaria cases. Similarly, in Lakki Marwat, the risk increased by 9.49% (95%CI: 5.25, 13.91) per 1 °C rise in temperature.

The risk of PV infections followed a comparable pattern in both locations. In Bannu, the risk of PV infections rose by an estimated 9.60% (95%CI: 4.50, 14.96) with temperature increments, mirroring the trend in Lakki Marwat, where it increased by 9.48% (95%CI: 5.21, 13.93).

However, when considering PF infections, the contribution to higher risk varied between the districts. In Bannu, a 1 °C increase above 22.4 °C was associated with a substantial 14.95% (95%CI: 6.07, 24.57) rise in PF infections. In contrast, Lakki Marwat displayed a lower association, with PF infection contributing to a 4.74% (95%CI: −2.96, 13.06) increase in risk.

Figures 4 and 5 further indicate that as the monthly lag increases from 0 to 3 months, there is a noticeable escalation in the risk of malaria cases, with two distinct peaks observed at lag 1 and lag 3. In Bannu, the risk is estimated to be 3.66% (95%CI: 0.00, 7.44) at lag 1 and 6.00% (95%CI: 2.77, 9.27) at lag 3. Similarly, in Lakki Marwat, the risk is estimated to be 6.28% (95%CI: 1.85, 10.91) at lag 1 and 7.14% (95%CI: 3.61, 10.80) at lag 3. This suggests that factors influencing malaria occurrence might have a delayed effect, becoming more influential several months after their initial occurrence.

### Projected association between temperature and malaria

The attributable fractions outlined in Table 3 provide compelling insights into the impact of rising temperatures on malaria cases. In Bannu, up to 39.76% (95%CI: 23.19, 51.93) of reported malaria instances are linked to temperatures ranging between 22.4 °C and 35.3 °C. Similarly, in Lakki Marwat, 54.05% (95%CI: 34.64, 68.82) of cases are attributable to this temperature range.

**Table 3.** Attributable fraction of malarial cases associated with mean temperatures in baseline period (2014–2022) and projected climate change scenarios (2044–2052 and 2064–2072) for Bannu and Lakki Marwat

City			Attributable Fraction PV	Attributable Fraction PF	Attributable Fraction Total Cases
Bannu		Baseline	39.1 (21.7, 51.7)	45.3 (24.1, 59.0)	39.8 (23.2, 51.9)
	2044–2052	SSP2 4.5	41.4 (23.4, 54.2)	51.3 (27.9, 65.8)	42.2 (25.0, 54.5)
		SSP5 8.5	42.3 (24.1, 55.0)	51.8 (28.4, 66.1)	43.0 (25.7, 55.3)
	2064–2072	SSP2 4.5	42.5 (24.2, 55.3)	52.5 (28.9, 66.9)	43.3 (25.8, 55.5)
		SSP5 8.5	39.9 (23.1, 51.2)	51.6 (28.9, 64.8)	40.6 (24.6, 51.6)
Lakki Marwat		Baseline	54.0 (34.4, 68.9)	28.8 (−32.0, 66.6)	54.1 (34.6, 68.8)
	2044–2052	SSP2 4.5	55.3 (35.7, 69.8)	30.9 (−35.9, 70.7)	55.4 (36.0, 69.8)
		SSP5 8.5	57.2 (37.3, 71.7)	32.5 (−38.8, 73.7)	57.3 (37.6, 71.8)
	2064–2072	SSP2 4.5	57.4 (37.5, 72.0)	32.6 (−39.1, 74.0)	57.6 (37.7, 72.0)
		SSP5 8.5	53.4 (35.2, 66.5)	33.1 (−40.9, 74.4)	53.7 (35.5, 66.7)

Projections based on the Shared Socio-Economic Pathways indicate a rise in the attributable fraction of heat-related malaria cases in both districts.

## Discussion

This study provides the first evidence from Pakistan by accounting for temporal and lagged dependencies in predicting the association between monthly temperature conditions and malaria incidence and projecting future trends. The results indicate a direct association between temperature and malaria incidence in the southern districts of KPK. Findings from this study suggest that temperatures exceeding 22.4 °C corresponded to a 9–10% increase in malaria transmission for every 1 °C rise. These findings are consistent with a recent study [22] from Bannu, Pakistan, suggesting that with every 1 °C increase in temperature, the percent variation in the odds ratio of malaria incidence increases by 4%. Similarly, research conducted in multiple Chinese provinces reported a 6.7–15.8% rise in malaria cases for every 1 °C increase in maximum temperature [23]. Several recent studies from developing countries, such as Iran and Uganda, discerned similar patterns [24–25]. Numerous studies indicate a rise in malaria transmission beyond specific temperature thresholds [26–27]. However, a few studies conducted in China and sub-Saharan Africa provide contrasting evidence in the context of climate change [28–29]. The conflicting results could be attributed to disparities in different modelling approaches [30]. Localized regional conditions play an important role in the transmission dynamics of malaria and should be considered when assessing the complex associations between environmental factors and malaria [31].

The relationship between temperature and malaria transmission tends to be intricate, influenced by multiple interacting variables such as humidity, rainfall patterns, other local environmental conditions, and human behaviour. In tropical countries, such as sub-Saharan Africa, where temperature remains consistently warm throughout the year, conducive conditions for the breeding and survival of malaria-carrying mosquitoes persist [32]. However, with reduced rainfall and hotter, drier conditions, the decrease in standing water and consequent breeding sites might temporarily reduce mosquito populations, thus lowering disease transmission. In subtropical monsoon-fed climates typical of Pakistan, temperature and rainfall patterns vary annually. Monsoon summer seasons can create periods of increased standing water, providing breeding grounds for mosquitoes. During these times, malaria transmission may surge due to the shortened development time of the malaria parasite and the availability of more habitats for mosquito breeding [1]. Other ecological factors, like topography, also play a crucial role in malaria transmission. For example, the flat terrain of Bannu and Lakki Marwat can lead to poor drainage of rainwater, resulting in standing stagnant water [12]. The presence of rivers, canals, and irrigation systems in and around southern districts of KPK, such as Bannu and Lakki Marwat, provide ample breeding places for mosquitoes and play a key role in mosquito ecology and malaria transmission [12]. Furthermore, since the 1930s, numerous irrigation channels in the Bannu cantonment have served as primary mosquito breeding sites for *Anopheles* species, such as *An. stephensi*, *An. culicifacies*, and *An. subpictus* [10]. Due to the endophilic behaviour of *Anopheles* in Pakistan, the species are generally found indoors [12]. In arid regions like Bannu and Lakki Marwat, water is often stored in open containers inside houses, which can serve as breeding sites for mosquitoes. Human factors such as poorly constructed housing, particularly in rural settings (e.g. thatched roofs and unsealed walls), can increase exposure to mosquito bites. Nearly a century ago, malaria surveys revealed that cases began to rise towards the end of August, reaching their peak in October and November, before declining rapidly [10]. Currently, peaks in malaria transmission are observed from September to December and April to May, which may be influenced by changing climatic patterns, including temperature, precipitation, variations in rainy seasons, and agricultural activities [7, 9, 12]. Extensive agricultural practices, particularly rice paddies and other water-intensive crops, as well as trade (particularly the used tire trade, which is quite common in KPK) and close contact with livestock in rural settings, can also facilitate malaria transmission. Many studies have highlighted the role used tires in the transmission of vector-borne disease [33–34]. Used tires, when stored, recycled, or discarded improperly, collect rainwater and create stagnant pools, providing ideal breeding sites for mosquitoes. In addition, limited access to healthcare facilities and resources, particularly for socioeconomically disadvantaged communities, can hinder prompt diagnosis and treatment, exacerbating the spread of malaria.

Furthermore, this study reveals a delayed impact of mean temperature on the burden of malaria, persisting for up to three months. The peak at a 1-month lag could be associated with the Extrinsic Incubation Period (EIP) of malaria parasites within mosquitoes and accelerated larval development. As temperatures rise, mosquitoes may become infectious more quickly, leading to increased malaria transmission within one month. For example, within the temperature range of 25–30 °C, the EIP for *P. vivax* in mosquitoes is likely around 8–10 days, whereas, below 20 °C, the EIP can extend up to 35 days [35]. The peak at 3-month lag suggests a more extended impact of temperature changes on the malaria transmission cycle. This could be due to several factors, including: (i) sustained high temperatures over a period, leading to an increased and continuous population of infectious mosquitoes [36], (ii) the compounding effect of multiple mosquito generations, where an initial temperature increase results in a progressively higher mosquito population over time [37], and (iii) Possible delays in human behaviour or environmental factors that influence mosquito breeding sites [38].

The increased overall risk can be linked to the faster reproduction rate triggered by warming temperatures, thereby extending the time frame for mosquito breeding. The observed effect of environmental covariates at each lag may represent a cumulative influence from preceding lags [25]. Additionally, recent extreme environmental conditions, such as prolonged rainy seasons, could also contribute to the impact on the malaria burden. This finding aligns with prior studies highlighting how disease risk temporally shifts in response to temperature variations. Importantly, an increase in temperature substantially amplifies the incidence rate of malaria, both in the current month and in subsequent months [24, 25, 37, 38]. The month-lagged effects of temperature offer a sufficient time frame for designing interventions to interrupt malaria transmission. These findings are crucial for administering institutes like IVC/MCP-KP and FPHC, which are responsible for malaria control in KPK, Pakistan. Building on their existing efforts in distributing bed nets, conducting IRS, and managing cases with RDTs and antimalarial treatments, a targeted approach could be employed considering the seasonal nature and lagged effect of temperature on malaria transmission. For instance, mass distribution of bed nets before the transmission season, IRS before the monsoon, and preventive treatment administration to vulnerable groups during peak periods could significantly reduce the malaria burden. Additionally, timely awareness campaigns and healthcare worker training can better prepare communities for early detection and prevention.

Some of the challenges that hinder effective malaria control in Pakistan include the misuse and overuse of antimalarial drugs, the use of substandard and counterfeit medications, limited access to healthcare and infrastructure – particularly in rural and remote areas – inadequate vector control programmes, lack of awareness about environmental factors that favour mosquito breeding, socioeconomic barriers, inadequate preparedness for climatic challenges and extreme weather conditions such as rainfall and floods, and overall political and financial instability in the country [39].

This study further suggests that the temperature-related attributable fraction of malaria cases is projected to increase from 39.8 to 43.3% and from 54.1 to 57.6%, for the two districts in projected scenarios (SSP2 4.5), as warmer temperatures become more frequent. These findings indicate the critical role of temperature in malaria transmission, also suggesting that climate change is likely to exacerbate malaria transmission. Pakistan already faces a high burden of malaria and other infectious diseases. An increase in malaria cases due to warming temperatures could lead to higher mortality and morbidity, further straining already overstretched healthcare systems and causing significant economic impacts on socioeconomically vulnerable communities [39]. Additionally, under high-emission scenarios (SSP2 8.5), the attributable fractions show a slight reduction, likely due to the frequent occurrence of extreme temperature conditions, which may not be conducive to malaria transmission.

Climate change is anticipated to exert both direct and indirect influences on the transmission of malaria, particularly affecting the most vulnerable communities. While there is limited data on the long-term ramifications of climate change on malaria transmission, recent events in Pakistan illustrate how extreme weather events, specifically floods, have led to a substantially increased burden of malaria. As is evident in this study, despite the gradual decline in malaria from 2014 onwards, a striking surge in malaria cases was observed in 2022. In that year, unprecedented flooding submerged one-third of the country, causing widespread devastation and health challenges, including a significant rise in malaria [1]. The WHO reported a more than four-fold increase in malaria cases in Pakistan in 2022 compared to 2021, totalling over 1.6 million cases [1].

Climate change is expected to impact malaria transmission across various scenarios, potentially affecting both traditionally endemic tropical regions and historically non-endemic areas, such as higher-altitude temperate zones [40]. Warmer temperatures can accelerate parasite growth cycles in mosquitoes, amplifying transmission rates and altering the overall burden of the disease. Shifts in temperature, rainfall, and humidity might expand the habitat range of malaria-carrying mosquitoes, leading to transmission in previously unaffected areas, such as the recent resurgence of malaria cases in Europe [40]. Conversely, an increase in heatwaves and extremely hot days may reduce malaria transmission in highly endemic areas. Temperature and humidity intricately influence mosquito life cycles, potentially increasing *Anopheles* mosquito frequency and biting rates while also shortening the extrinsic incubation period of *Plasmodium* parasites [40]. This study provides compelling evidence of how temperature changes likely contributed to malaria transmission in vulnerable regions.

Further, the broader impacts of climate change – such as adverse health effects and socioeconomic setbacks – can impede disease control efforts, potentially fostering increased malaria transmission. Vulnerable populations in low and middle-income countries facing economic hardship and limited access to healthcare are particularly susceptible. The compounding impacts of rising temperatures and more frequent extreme weather events, such as floods, significantly influence malaria prevalence and transmission. For example, during the 2022 floods in Pakistan, extreme flooding displaced individuals from their residences, exposing them to mosquito-infested environments. The stagnant water left behind created optimal breeding grounds for mosquitoes, persisting for extended periods. Additionally, the inundation adversely affected healthcare facilities and disrupted transportation, leaving those afflicted with illness without access to treatment.

Climate change poses a threat to the intricate interplay between natural and human systems, undermining various social determinants of good health, including livelihoods, nutrition, security, and access to quality health services. In countries like Pakistan, facing the compounded challenges of infectious diseases and climate change, urgent evidence-based studies are crucial for crafting targeted policies. These studies need to focus on the intricate relationship between changing climate patterns and malaria transmission within specific localities. By establishing robust surveillance systems, fostering community engagement, and integrating climate-resilient strategies into healthcare infrastructure, policymakers can adaptively address the evolving risks. Cross-sector collaborations and investment in capacity building will be pivotal in ensuring a comprehensive approach that not only targets malaria control but also strengthens resilience against the health impacts of a changing climate.

### Limitations

This study provides empirical evidence regarding the impacts of rising temperatures on malaria transmission in two endemic districts of Pakistan and highlights the influence of climate change on malaria dynamics. However, several limitations in the study design should be noted.

Firstly, this study relied on temperature data from global gridded meteorological datasets to measure temperature exposure. While this method may overlook micro-scale spatial and temporal variations in temperature that are critical for mosquito distribution, it has been successfully used in previous studies to quantify the impacts of meteorological conditions on malaria transmission [25].

Secondly, analyzing monthly average temperatures may obscure diurnal or daily temperature variability, potentially masking the immediate impact of temperature fluctuations on mosquito ecology and malaria transmission.

Thirdly, this study did not account for all possible environmental covariates, such as altitude, land-use practices like agriculture, or vector distribution, which could influence malaria transmission.

Fourth, malaria cases were diagnosed using both RDTs and microscopy. While microscopy is the gold standard, RDTs were used in several locations due to resource constraints and cost considerations, posing a risk of including false-positive cases. However, previous studies in the region have shown significant agreement between microscopy and RDT results [6]. Therefore, data from both sources were integrated to provide a comprehensive view of malaria prevalence.

Despite these limitations, this study offers valuable population-level insights crucial for understanding the current and projected impacts of temperature on malaria transmission dynamics. Future research should prioritize integrating more detailed microclimatic data to better quantify the influence of temperature and relative humidity on vector density. Conducting vector sampling across diverse regions and seasons is essential to develop a comprehensive spatiotemporal profile of vector distribution, providing critical evidence for targeted malaria control interventions.

## Conclusions

Changing climatic patterns and consequent extreme weather events are associated with malaria resurgence in Pakistan. It is imperative to estimate the climate change-attributed increase in the risk of malaria in vulnerable regions of Pakistan. This study provides evidence showing a direct association between monthly temperature conditions and malaria incidence in the southern districts of KPK, Pakistan. Specifically, temperatures exceeding 22.4 °C correspond to a 9–10% increase in malaria transmission for every 1 °C rise. The relationship between temperature and malaria transmission is intricate and is influenced by several factors such as precipitation, humidity, and other local environmental conditions and human behaviour. The study reveals a lagged impact of mean temperature on malaria incidence, persisting for up to three months. This lagged effect is likely due to the Extrinsic Incubation Period (EIP) of malaria parasites within mosquitoes and accelerated larval development. The lagged effects of temperature on malaria provide a sufficient timeframe for designing interventions to interrupt malaria transmission. Effective malaria control measures include vector control, chemoprevention, case management, surveillance, and community engagement, such as community health education campaigns. The attributable fraction of malaria cases associated with higher temperatures is projected to increase. Rising temperatures and extreme weather events, such as the 2022 floods in Pakistan, have already shown a significant impact on malaria incidence. Urgent, evidence-based studies are needed to craft targeted policies addressing the interplay between climate change and malaria transmission. Policymakers should focus on establishing robust surveillance systems, fostering community engagement, and integrating climate-resilient strategies into healthcare infrastructure.

## Data Availability

Malaria Data were acquired from the Integrated Vector Control/Malaria Control Program, KPK, Pakistan upon request. These datasets are not publicly available but can be obtained from the data custodians in the Directorate General of Health Services, KPK, Pakistan. Environmental data were acquired from Copernicus ERA5-Land monthly averaged data, which are publicly available and can be downloaded from the following link: https://cds.climate.copernicus.eu/cdsapp#!/dataset/reanalysis-era5-land-monthly-means?tab=overview. All relevant R codes used for the analyses and results are publicly available at https://doi.org/10.5281/zenodo.14751632

## References

[1] World Health Organization (2023) World Malaria Report 2023. https://www.who.int/publications/i/item/9789240086173 (accessed 19 November 2024).

[2] Centers for Disease Control and Prevention (n.d.) Impact and Burden of Malaria. https://www.cdc.gov/malaria/php/impact/index.html (accessed 19 November 2024)

[3] Parham PE, Michael E (2010) Modeling the effects of weather and climate change on malaria transmission. Environmental Health Perspectives 118, 620–626. 20435552 10.1289/ehp.0901256 PMC2866676

[4] Pascual M, et al. (2008) Shifting patterns: Malaria dynamics and rainfall variability in an African highland. Proceedings of the Royal Society B: Biological Sciences 275, 123–132. 10.1098/rspb.2007.1068 PMC2596179 17999952

[5] Rogers DJ (1996) Changes in disease vector distributions. In Hulme M (ed.), Climate Change and Southern Africa: An Exploration of Some Potential Impacts and Implications in the SADC Region. Norwich: Climate Research Unit, University of East Anglia, pp. 49–55.

[6] Jahan F, et al. (2019) Malaria epidemiology and comparative reliability of diagnostic tools in Bannu; an endemic malaria focus in south of Khyber Pakhtunkhwa, Pakistan. Pathogens and Global Health 113, 75–85. 30894081 10.1080/20477724.2019.1595904 PMC6493316

[7] Khan MI, et al. (2023) Malaria prevalence in Pakistan: A systematic review and meta-analysis (2006–2021). Heliyon 9(4), e15373. 37123939 10.1016/j.heliyon.2023.e15373 PMC10133748

[8] Barraud PJ (1934) The Fauna of British India, Including Ceylon and Burma. Diptera Vol 5, Family Culicidae. Tribe Megarhinini and Culicini. London: Taylor and Francis; 463p.

[9] Khan MA (2022) Seasonal variation in species composition and relative abundance of mosquitoes in the district Bannu, Khyber Pakhtunkhwa, Pakistan: Prospects of dengue fever in the study area. International Journal of Tropical Insect Science 42, 2221–2231.

[10] Dogra JR (1938) A Malaria Survey of Bannu Cantonment (1932-33). Journal of the Malaria Institute of India 1(1), 57–81.

[11] Reisen WK, Azra K, Mahmood F (1982) Anopheles culicifacies (Diptera: Culicidae): Horizontal and vertical estimates of immature development and survivorship in rural Punjab Province, Pakistan. Journal of Medical Entomology 19, 413–422. 7154019 10.1093/jmedent/19.4.413

[12] Herrel N, et al. (2004) Adult anopheline ecology and malaria transmission in irrigated areas of South Punjab, Pakistan. Medical and Veterinary Entomology 18, 141–152. 15189239 10.1111/j.0269-283X.2004.00481.x

[13] Ejov M (2020) National Strategic Plan for Malaria Elimination in Pakistan 2021–2035. https://www.apmen.org/sites/default/files/all_resources/Pakistan%20National%20Strategic%20plan%20%28NSP%20MCE%202021-2035%29.pdf

[14] Mukhtar EM (ed.) (2004) Economic Analysis for a National Study on Malaria Control in Pakistan. Islamabad: Malaria Control Programme, Ministry of Health.

[15] Sabater MJ (2019) ERA5-Land monthly averaged data from 1950 to present. Copernicus Climate Change Service (C3S) Climate Data Store (CDS) [Online]. 10.24381/cds.68d2bb30. (accessed 22 March 2024).

[16] Lawrence MG (2005) The relationship between relative humidity and the dewpoint temperature in moist air: A simple conversion and applications. Bulletin of the American Meteorological Society 86(2), 225–234.

[17] Gasparrini A, Armstrong B, Kenward MG (2010) Distributed lag non-linear models. Statistics in Medicine 29(21), 2224–2234. 20812303 10.1002/sim.3940 PMC2998707

[18] Suh E, et al. (2020) The influence of feeding behaviour and temperature on the capacity of mosquitoes to transmit malaria. Nature Ecology & Evolution 4, 940–951. 32367033 10.1038/s41559-020-1182-x PMC7334094

[19] Gasparrini A, Leone M (2014) Attributable risk from distributed lag models. BMC Medical Research Methodology 14, 1–8. 24758509 10.1186/1471-2288-14-55 PMC4021419

[20] World Bank Group. Climate Change Knowledge Portal. https://climateknowledgeportal.worldbank.org/ (accessed 3 December 2023).

[21] Gasparrini A (2011) Distributed lag linear and non-linear models in R: The package dlnm. Journal of Statistical Software 43, 1–20. PMC3191524 22003319

[22] Haq IU, et al. (2024) Modeling the effect of climatic conditions and topography on malaria incidence using Poisson regression: A retrospective study in Bannu, Khyber Pakhtunkhwa, Pakistan. Frontiers in Microbiology 14, 1303087. 38287956 10.3389/fmicb.2023.1303087 PMC10822983

[23] Xiang J, et al. (2018) Association between malaria incidence and meteorological factors: A multi-location study in China, 2005–2012. Epidemiology & Infection 146, 89–99. 29248024 10.1017/S0950268817002254 PMC9134552

[24] Mohammadkhani M, et al. (2016) The relation between climatic factors and malaria incidence in Kerman, south east of Iran. Parasite Epidemiology and Control 1, 205–210. 29988199 10.1016/j.parepi.2016.06.001 PMC5991842

[25] Okiring J, et al. (2021) Associations between environmental covariates and temporal changes in malaria incidence in high transmission settings of Uganda: A distributed lag nonlinear analysis. BMC Public Health 21, 1–11. 34717583 10.1186/s12889-021-11949-5 PMC8557030

[26] Mordecai EA, et al. (2013) Optimal temperature for malaria transmission is dramatically lower than previously predicted. Ecology Letters 16, 22–30. 23050931 10.1111/ele.12015

[27] Sehgal M, Ghosh S (2020) Exploring the usefulness of meteorological data for predicting malaria cases in Visakhapatnam, Andhra Pradesh. Weather, Climate, and Society 12, 323–330.

[28] Hay SI, et al. (2002) Climate change and the resurgence of malaria in the east African highlands. Nature 415, 905–909. 11859368 10.1038/415905a PMC3164800

[29] Wang Z, et al. (2022) The relationship between rising temperatures and malaria incidence in Hainan, China, from 1984 to 2010: A longitudinal cohort study. The Lancet Planetary Health 6, e350–e358. 35397223 10.1016/S2542-5196(22)00039-0

[30] Beloconi A, et al. (2023). Malaria, climate variability, and interventions: Modelling transmission dynamics. Scientific Reports 13(1), 7367. 37147317 10.1038/s41598-023-33868-8 PMC10161998

[31] Babaie J, et al. (2018). A systematic evidence review of the effect of climate change on malaria in Iran. Journal of Parasitic Diseases 42, 331–340. 30166779 10.1007/s12639-018-1017-8 PMC6104236

[32] Fall P, et al. (2023) Enhancing understanding of the impact of climate change on malaria in West Africa using the vector-borne disease community model of the International Center for Theoretical Physics (VECTRI) and the Bias-corrected phase 6 coupled model Intercomparison project data (CMIP6). Microbiology Research 14, 2148–2180.

[33] Reiter P, Sprenger D (1987). The used tire trade: a mechanism for the worldwide dispersal of container breeding mosquitoes. Journal of American mosquito control association 3(3), 494–501. 2904963

[34] Fatima SH, et al. (2018). Patterns of occurrence of dengue and chikungunya, and spatial distribution of mosquito vector Aedes albopictus in Swabi district, Pakistan. Tropical Medicine & International Health 23(9), 1002–1013. 29956428 10.1111/tmi.13125

[35] Queensland Health (2011) Guideline for the Management of Community Outbreaks and Epidemics of Malaria in Torres Strait. https://www.health.qld.gov.au/__data/assets/pdf_file/0022/444325/malaria-gl.pdf (accessed 19 June 2024).

[36] Paaijmans KP, et al. (2010). Influence of climate on malaria transmission depends on daily temperature variation. Proceedings of the National Academy of Sciences 107(34), 15135–15139. 10.1073/pnas.1006422107 PMC2930540 20696913

[37] Beck-Johnson LM, et al. (2013). The effect of temperature on Anopheles mosquito population dynamics and the potential for malaria transmission. PLoS One, 8(11), e79276. 24244467 10.1371/journal.pone.0079276 PMC3828393

[38] Zhao, X., et al (2014). Characterizing the effect of temperature fluctuation on the incidence of malaria: An epidemiological study in South-West China using the varying coefficient distributed lag non-linear model. Malaria Journal 13, 1–10. 24886630 10.1186/1475-2875-13-192 PMC4050477

[39] Vohra LI, et al. (2023). Rising cases of dengue and malaria in flood affected areas of Pakistan: A major threat to the Country’s healthcare system. Disaster Medicine and Public Health Preparedness 17, e323. 36789658 10.1017/dmp.2022.293

[40] Fischer L, et al. (2020). Rising temperature and its impact on receptivity to malaria transmission in Europe: A systematic review. Travel Medicine and Infectious Disease 36, 101815. 32629138 10.1016/j.tmaid.2020.101815

